# Frequent high-risk HPV co-infections excluding types 16 or 18 in cervical neoplasia in Guadeloupe

**DOI:** 10.1186/s12885-021-07940-3

**Published:** 2021-03-16

**Authors:** Stanie Gaete, Aviane Auguste, Bernard Bhakkan, Jessica Peruvien, Cecile Herrmann-Storck, Youri Socrier, Abdoulaye Diedhiou, Jacqueline Deloumeaux

**Affiliations:** 1 Biological Resources Center Karubiotec™, BRIF n° KARUBIOTEC-GUA-00971, Pointe-à-Pitre, Guadeloupe; 2 Guadeloupe cancer registry, University Hospital of Guadeloupe, Pointe-à-Pitre, Guadeloupe; 3 Virology-Microbiology Unit, University Hospital of Guadeloupe, Pointe-à-Pitre, Guadeloupe; 4 Pathology Laboratory ALIZES, Baie-Mahault, Guadeloupe; 5 Pathology Laboratory, University Hospital of Guadeloupe, Pointe-à-Pitre, Guadeloupe

**Keywords:** Human papilloma virus, Cervical cancer, Caribbean, Guadeloupe

## Abstract

**Background:**

Cervical cancer is the fourth cancer worldwide. The Human Papilloma Virus is responsible for 99% of the cases but the distribution of its genotypes varies among populations. We aimed to identify HPV genotypes distribution in women with grade 2/3 cervical intraepithelial dysplasia or invasive cervical cancer in Guadeloupe, a French Caribbean territory with a population mainly of African descent.

**Methods:**

We used paraffin-embedded tumors for viral DNA extraction from women diagnosed between 2014 and 2016 and identified by the population-based cancer registry. The HPV Genotyping was performed with the InnoLIPA HPV Genotyping Extra kit®.

**Results:**

Overall, 213 samples out of the 321 eligible records were analyzed. The HPV status was positive for 94% of the cases. The five most common oncogenic HPV genotypes were HPV31 (47%), HPV33 (38%), HPV16 (32%), HPV44 (31%) and HPV26 (28%). HPV18 was found in only in 5% of the cases. Among the studied cases, 94% had multiple infections. More than 60% of single infections were HPV16-related, accounting for 35% of HPV16 infections.

**Conclusions:**

These results show a different distribution of oncogenic HPVs in Guadeloupe with “31 >  33 > 16” and a high frequency of multiple infections. Despite a lower coverage, the nine-valent vaccine is nevertheless adequate.

## Background

Cervical cancer is the 4th cancer worldwide, accounting for 3.2% of all new cancer cases and for 7.5% of cancer deaths in women in 2018 [1]. The incidence of this cancer varies by region. It is lower in developed countries that have been using for many years the cervical smear test as a screening tool. The Caribbean and South America are among the regions with the highest incidence rates [2]. However, over the period 2008–2014, the two French departments of Guadeloupe and Martinique registered incidence rates of 8.7 and 7.2 per 100,000 persons/year, which were lower than that of their Caribbean neighbours but greater than rates in France and other European Countries [3].

The Human Papilloma Virus (HPV) is responsible for the development of precancerous and cancerous cervical lesions, and up to 99% of cervical cancers are estimated to be HPV related [4]. However, a variation of HPV genotypes distribution is described [5] and to date, more than 200 genotypes have been identified. Among them, about 40 can infect the genital tract and 13 are considered to be of high oncogenic risk in humans [6]. Types 16 and 18 are respectively responsible for 54.4 and 16.5% of cases worldwide [7, 8]. However, it has been shown that the genotypic distribution of oncogenic HPV in patients of African descent may differ from that found in Europe and north America [7–11].

Cervical cancer screening is implemented in Martinique since 1991 as part of national population-based pilot programs conducted in four regions [12]. Despite a decreasing trend documented by the cancer registry [3], the incidence rate of invasive cervical cancer remains higher than France. In Guadeloupe, there is no organized screening, but women have access to individual screening covered by the national health insurance.

The HPV vaccine is available since 2015 in both territories and is recommended for young girls under 14 years. Unfortunately, vaccination coverage remains very low in both Guadeloupe and Martinique. In 2018, the full HPV vaccination coverage among girls at 16 years old was 11.9% in Guadeloupe and 8.2% in Martinique [13]. The population’s mistrust of vaccination may be fueled by discussions on the effectiveness of the HPV vaccines and their adequacy for our populations.

Indeed, national policies for cervical cancer prevention may not be appropriate for the Guadeloupean context. Recent work among healthy Guadeloupean women [14] has already demonstrated a high proportion of high-risk HPV (HR-HPV) types excluding HPV16 and 18 (28.8%) which differs considerably to what is known in the mainland France [15]. This particularity warrants adaptations for regional policies; however, to date, the HPV genotype distribution among women with cervical lesions and invasive cervical cancer has not been studied.

The objective of this study was to determine the prevalence of circulating HPV genotypes in cervical cancers in Guadeloupe using paraffin embedded blocks of tissue from cervical tumours.

## Methods

### Study samples

A retrospective study was conducted on women with invasive cervical cancer or high grade cervical intraepithelial neoplasm (CIN 2/3) identified by the cancer registry between 2014 and 2016. Eligible participants’ tumour blocks and slides for which informed consent was obtained were included in the study. Ethics approval was granted by the local ethics committee of the University hospital of Guadeloupe (approval n° A21_02_05_17)**.**

Paraffin-embedded blocks containing cervical tumour tissue were accessed from the only two existing pathology labs in Guadeloupe. The blocks were de-archived, re-read by the referent pathologists to mark-off the tumor rich zone on the slides, and sent to Karubiotec™, the Biological resource center of University Hospital of Guadeloupe for processing. Karubiotec’s™ activities consist of receiving, conserving, and ensuring the availability of human biological resources. It is authorized by the French Ministry of Education, Higher Education and Research and is approved by a national research ethics committee, under the numbers AC-2018-3313, and DC-2013-1941 modified. Karubiotec™ is also dually certified according to NF S 96–900 and ISO 9001 standards under the number 150197/1289F. The dedicated software used for the management of biological collections, ModulBio®, is declared to the French Data protection authority, CNIL, under the number 1682116v0.

The HPV genotyping carried out on the Karubiotec™ molecular biology platform was validated by the expert microbiologist of the University Hospital of Guadeloupe. For each person with identified material, a 5-10 μm thick section block was extracted, in accordance with the richest zone of tumor cells marked by the pathologist.

### Deparaffinization process

Before processing for DNA extraction, the successive steps were performed: 1) Incubation at 56 °C for 3 min with 320 μL of deparaffinization solution (cat/ID19093; QIAGEN SA); 2) Reduction of the temperature down to 15–25 °C 3) Addition of 180 μL of ATL Buffer and mixing by vortexing; 4) Centrifugation 1 min at 11000 g; 5) Addition of 20 μl proteinase K to the lower phase and mixing by pipetting up and down; 6) Incubation 1 h at 56 °C; 7) New 1 h incubation at 90 °C.

### DNA extraction and HPV genotyping

After the last incubation, and centrifugation to remove drops on the lid, the lower clear phase was transferred into a new tube to continue the DNA purification step, with the QIAamp DNA FFPE tissue kit (cat/ID56404; QIAGEN SA). All samples were processed according to manufacturer’s protocols and were eluted in a 100 μL final ATE Buffer. For all DNA, Optical Density (OD) was measured for Quality Control with OD260nm/ OD280nm ratio on a nanodrop® One (Thermofisher®).

The HPV Genotyping was performed with the InnoLIPA HPV Genotyping Extra kit® (ID 81533: amplification kit and ID 81534: hybridization kit) [16]. In this protocol 10 μL of each DNA was amplified with SPF10 primers to detect a part of L1 region in the HPV genome.

In the same reaction tube, 2 other primers specific to the HLADPB1 gene provided supplementary quality control for the DNA extraction. All probes were first immobilized on a membrane strip. After hybridization, stringent washes, addition of alkaline phosphatase conjugated streptavidin and incubation with BCIP/NBT chromogen, the revealed parallel lines corresponded to:

- Quality controls of extraction and hybridization,
- HPV High risk genotypes (HR-HPV): HPV16 HPV18 HPV31 HPV33 HPV35 HPV39 HPV45 HPV51 HPV52 HPV56 HPV58 HPV59 HPV68.
- HPV potentially High-risk genotypes (pHR-HPV): HPV26 HPV53 HPV66 HPV70 HPV73 HPV82
- HPV Low risk and non-categorized genotypes (LR-HPV): HPV06 HPV11 HPV42 HPV40 HPV43 HPV44 HPV54 HPV61 HPV62 HPV67 HPV81 HPV83 HPV89.

Each technical run had a positive and negative control.

For 25 patients with negative HPV tests, the data collected for P16 and Koilocytes from the pathology reports were used to confirm the negative result. Out of the 25 patients, 13 had insufficient tumor materials for HPV genotyping but were positive for either P16 of Koilocytes. P16 overexpression is a surrogate biomarker of HPV infection with a high specificity to detect high grade dysplasia [17, 18]. HPV infection of the squamous cervix also determine the incidence of koilocytes in cervical smears and biopsies [18]. The data were excluded from the genotype analyses but kept in the descriptive analysis study. For the 12 remaining cases, HPV status could be neither attributed based on PCR test nor pathology report. We therefore considered these patients as negative for HPV.

### Data collection

Clinical and demographic data was extracted from the Guadeloupe cancer registry database which has been recording incident cancer cases since 2008. The registry is part of the national program for epidemiological surveillance of cancer in France, within the French network of cancer registries (FRANCIM). International and standardized data collection and coding procedures with International Classification of Diseases for Oncology (ICD-O3) are used in compliance with International Agency for Research on Cancer (IARC) and European Network of Cancer Registries (ENCR). For cervical cancer, we use the revised IARC/WHO 2014 classification. The pathology reports for all cases are collected in our database and the diagnosis is based on primary histology in more than 95% of cases. High grade cervical intraepithelial neoplasms (CIN 2/3) are characterized by at least more than 2/3 of the epithelium, or the entire epithelium, replaced by abnormal cells.

This Registry is authorized by the National Authority for the protection of privacy and personal data (Commission Nationale de l’Informatique et des Libertés, CNIL). For each case, the data collected are: date of birth, date of diagnosis, and tumor characteristics (topography, histology, stage at diagnosis). Place and date of first treatment and type of treatment are also recorded.

### Statistical analyses

Descriptive analyses were performed to compare patients’ characteristics at diagnosis between histological grades, age groups and HPV status. The prevalence of each HPV genotype (in single or multiple concurrent infections) was given for the overall population, by histological grade and by age group. Our combinations were classified as HR/LR/pHR independently of their genotype. The resulting classification yielded 48 combinations involving between 1 and 6 HR types. We further classified these combinations into three categories according to the number of HR types involved: 1 HR, 2–3 HR and more than 3 HR.

The quantitative variables are presented as means (accompanied by their standard deviation) and qualitative variables as frequencies (percentage). Chi-squared test and Student t-test were used to assess the association between variables. When the assumptions for Chi-squared test were not met, an Exact Fisher test was performed. *P* values were considered significant under 0.05. All analyses were performed with R software version 3.6.0.

## Results

**Fig. 1:**
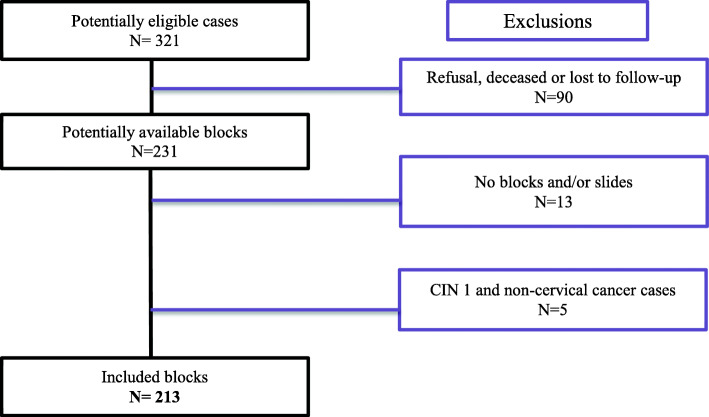
Flow chart for the constitution of our study sample

Figure 1 shows the selection procedure for the study. Overall, 213 subjects were studied, including 184 (86%) CIN 2/3 grade cases and 29 (14%) invasive cervical cancer cases. The mean age of our population was 41.5 years old (±13.5). It was higher in women with invasive cancer compared to those with CIN 2/3 (54.2 ± 16.5 vs 39.5 ± 11.6; *p = 0.0001*).

Table 1 presents HPV infection characteristics by histological grade and age group. An HPV-positive test was found for 94% of the tumours blocks, without significant difference between grades or age groups. We observed an infection with 2 or more virus in 154 (81.9%) of the HPV positive cases. Multiple infections involving 2 or 3 viruses were more frequently found in invasive cancer than in CIN 2/3 (65.4% vs 30.2%) but no differences was found for the number of infections between age groups.

**Table 1:** Sociodemographic, clinical characteristics and HPV profile of cases by histological grade and age group (Guadeloupe, 2014–2016)

	Overall	CIN 2/3 grades	Invasive cancers	*p*	< 40 years	41–50 years	> 50 years	*p*
	***n*** = 213	***n*** = 184	***n*** = 29		***n*** = 111	***n*** = 56	***n*** = 46	
Year of diagnosis, n(%)				0.53				0.25
2014	98 (46)	82 (45)	16 (55)		44 (40)	27 (48)	27 (59)	
2015	50 (23)	45 (24)	5 (17)		29 (26)	14 (25)	7 (15)	
2016	65 (31)	57 (31)	8 (28)		38 (34)	15 (27)	12 (26)	
HPV status, n(%)				0.67				0.33
HPV-	12 (6)	10 (5)	2 (7)		6 (5)	5 (9)	1 (2)	
HPV+	201 (94)	174 (95)	27 (93)		105 (95)	51 (91)	45 (98)	
	***n*** **= 188**	***n*** **= 162**	***n*** **= 26**		***n*** **= 97**	***n*** **= 48**	***n*** **= 43**	
Profile of infection^a^, n(%)				0.002				0.99
1 virus	34 (18.1)	32 (19.7)	2 (7.7)		18 (18.6)	9 (18.7)	7 (16.3)	
2 to 3 virus	66 (35.1)	49 (30.2)	17 (65.4)		33 (34.0)	17 (35.4)	16 (37.2)	
> 3 virus	88 (46.8)	81 (50.0)	7 (26.9)		46 (47.4)	22 (45.8)	20 (46.5)	
HPV oncogenicity risk^a^, n(%)								
High-risk	183 (91)	157 (91)	26 (93)	0.77	93 (84)	48 (86)	42 (91)	0.48
Probable High risk	104 (52)	90 (52)	14 (50)	0.95	52 (47)	27 (48)	25 (54)	0.69
Low risk and others	101 (51)	84 (49)	17 (61)	0.92	52 (47)	24 (43)	25 (54)	0.50
HR-HPV in combination^a^, n(%)				0.6				0.7
No HR	5 (2.7)	5 (3.1)	0		4 (4.1)	0	1 (2.3)	
1 HR	58 (30.8)	48 (29.6)	10 (38.5)		28 (28.9)	17 (35.4)	13 (30.2)	
2–3 HR	91 (48.4)	80 (49.4)	11 (42.3)		45 (46.4)	23 (47.9)	23 (53.5)	
> 3 HR	34 (18.1)	29 (17.9)	5 (19.2)		20 (20.6)	8 (16.7)	6 (13.9)	

^a^ Proportions are calculated after excluding positive cases with missing data for genotypes and negative cases

The frequencies of HR-HPV, pHR-HPV and LR-HPV did not differ between histological grades or age groups. HR-HPV was found in 91% in the overall population whereas pHR-HPV and LR-HPV were found respectively in 52% and 51% of all cases.

We analyzed the number of HR-HPV genotypes, regardless of the pHR or LR-HPV status. The most frequent combinations of HR-HPV (i.e. 2 to 3) was found in 48.4% of the overall samples without any difference between histological grades or age groups.

### Genotypes distribution

Table 2 shows HR-HPV distribution by histological grade. The 10 most common genotypes in the overall population were: HPV31 (47%), HPV33 (38%), HPV16 (32%), HPV44 (31%), HPV26 (28%), HPV52 (21%), HPV58 (20%), HPV56 (15%), HPV66 (15%) and HPV54 (13%). The most frequent genotypes for invasive cancer cases were HPV16 (38%) followed by HPV31 (35%). Likewise, the genotype distribution was fairly similar in the CIN 2/3 group, but HPV33 (40%) and HPV31 were the leading genotypes. HPV18 accounted for only 5% in the overall population without any difference between invasive cancer and CIN 2/3 cases. Among the low-risk genotypes, HPV06 was significantly associated with invasive cancers (*p* = 0.01).

**Table 2:** HPV genotype distribution among cases by histological grade (Guadeloupe, 2014–2016)

	Overall	CIN 2/3 grades	Invasive cancers	***p value***
	n = 188	n = 162	n = 26	
**High risk, n(%)**				
HPV31	88 (47)	79 (49)	9 (35)	*0.18*
HPV33	72 (38)	64 (40)	8 (31)	*0.39*
HPV16	61 (32)	51 (32)	10 (38)	*0.48*
HPV52	40 (21)	38 (23)	2 (8)	0.07
HPV58	37 (20)	31 (19)	6 (23)	*0.64*
HPV56	29 (15)	22 (14)	7 (27)	*0.08*
HPV68	20 (11)	18 (11)	2 (8)	*0.60*
HPV35	17 (9)	14 (9)	3 (12)	*0.63*
HPV51	16 (9)	12 (7)	4 (15)	*0.18*
HPV45	15 (9)	14 (9)	1 (4)	*0.40*
HPV39	14 (7)	11 (7)	3 (12)	*0.39*
HPV18	9 (5)	7 (4)	2 (8)	*0.45*
HPV59	7 (4)	5 (3)	2 (8)	*0.25*
**Potentially High risk, n(%)**				
HPV26	52 (28)	48 (30)	4 (15)	*0.13*
HPV66	29 (15)	24 (15)	5 (19)	*0.56*
HPV73	18 (10)	14 (9)	4 (15)	*0.28*
HPV82	18 (10)	13 (8)	5 (19)	*0.07*
HPV70	7 (4)	7 (4)	0	*0.28*
HPV53	2 (1)	2 (1)	0	*0.57*
**Low risk, n(%)**				
HPV44	58 (31)	52 (32)	6 (23)	*0.36*
HPV54	24 (13)	18 (11)	6 (23)	*0.09*
HPV06	21 (11)	14 (9)	7 (27)	*0.01*
HPV42	10 (5)	9 (6)	1 (4)	*0.72*
HPV61	6 (3)	6 (4)	0	*0.32*
HPV43	1 (1)	1 (1)	0	*0.69*
**Uncategorized risk, n(%)**				
HPV67	15 (8)	14 (9)	1 (4)	*0.40*
HPV62	5 (3)	5 (3)	0	*0.36*
HPV81	4 (2)	4 (2)	0	*0.42*
HPV89	3 (2)	3 (2)	0	*0.48*

Genotyping distribution is given for the 188 blocks for which an HPV genotype was found

### Combination distribution

The most frequent combination involving 3 viruses were HPV26–31-52 and HPV26–31-33. The association of HPV06 with invasive cancer was based on the consistency of its association with HPV16 (33% of the cases). Table 3 presents the distribution of HR-HPV combinations by histological grade stratified by the number of concurrent infections. Among patients with a single infection, HPV16 was the only genotype involved in invasive cancers and was present in 69% of the CIN 2/3 cases. For the latter, HPV35 was also found in 24% of the cases.

**Table 3:** Distribution of HR-HPV combinations by histological grade stratified by the number of concurrent infections (Guadeloupe, 2014–2016)

	1 virus infection			2–3 virus infections			> 3 virus infections		
	CIN2/3	Invasive cancers		CIN2/3	Invasive cancers		CIN2/3	Invasive cancers	
	N = 32	N = 2	*p*	*n* = 49	*n* = 17	*p*	*n* = 81	*n* = 7	*p*
**HR-HPV combinations, n(%)**									
1HR	29	2		19	8		0	0	
HPV16	20 (69)	2 (100)	0.28	11 (22)	4 (24)	1	0	0	
HPV33	1 (3.4)	0 (0)	0.80	0	0				
HPV35	7 (24)	0 (0)	0.45	1 (2)	2 (12)	0.16	0	0	
HPV51	1 (3.4)	0 (0)	0.80	5 (4)	2 (12)	1	0	0	
HPV56	–	–	–	2 (4)	0 (0)	1	0	0	
2 or 3 HR	–	–		29	9		51	2	
HPV16 + 1 other HR *	–	–	–	5 (10)	1 (6)	1	3 (4)	0 (0)	1
HPV[18–56]	–	–	–	1 (2)	2 (12)	0.16	2 (2)	0 (0)	1
HPV[31–33] + 1 other HR	–	–	–	4 (8)	2 (12)	0.64	32 (40)	1 (14)	0.25
HPV [31–52] + 1 other HR	–	–	–	14 (29)	1 (6)	0.09	6 (7)	1 (14)	0.45
HPV[39–45] + HPV68	–	–	–	2 (4)	1 (6)	1	2 (2)	0 (0)	1
More than 3 HR	–	–	–	–	–	–	29	5	
HPV[31–33] + 2 or more other HR *	–	–	–	–	–	–	19 (23)	4 (57)	0.07
HPV{16–52] + 2 or more other HR *	–	–	–	–	–	–	4 (5)	0 (0)	1
HPV[39–68] + 2 or more other HR*	–	–	–	–	–	–	4 (5)	0 (0)	1
HPV[33–52–58-68]	–	–	–	–	–	–	3 (4)	0 (0)	1

Other HR-HPV found in association with theses main combinations, involved 2 or 3 of the following HPV genotypes: 18, 35, 45, 51, 56, 58, 59, 68

Among patients infected with 2 or 3 viruses but with only one high-risk genotype, HPV16 remained the most frequently involved, both in CIN 2/3 and invasive cancer (respectively 22% and 24%). When in combination of 2 or 3, the most frequent HR-HPV association involved was HPV31–52 (respectively 29% of CIN 2/3 and 6% of invasive cancer), and to a lesser extent HPV31–33.

**Fig. 2:**
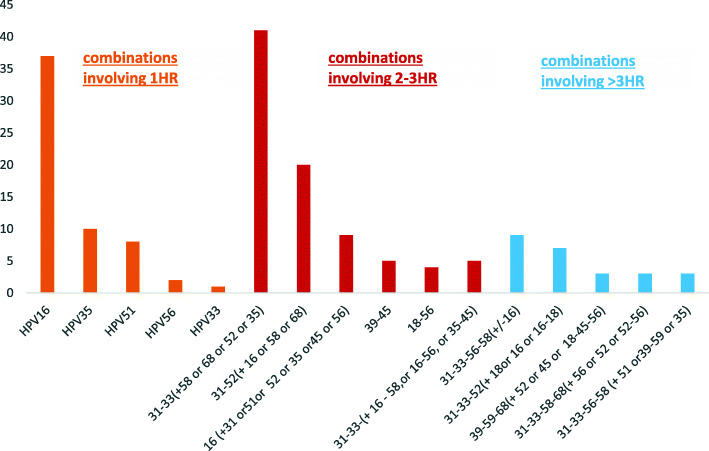
Most prevalent HR-HPV combinations involving multiple concurrent infections

For patients infected with more than 3 viruses, no single HR-HPV was found. In the different combinations, HPV31 along with either HPV52 (*n* = 14) or HPV33 (*n* = 32) were the most frequent high-risk genotypes involved. HPV31 and HPV33 (i.e. HPV 26–31-33 or, HPV 31–33) were involved in more than 60% of the cases. A combination involving 31–33–56-68 was found in invasive cancer among patients with more than 3 concurrent infections (*p* = 0.01). The most frequent HR-HPV combinations are shown in Fig. 2.

## Discussion

In this study performed on paraffin blocks from women with cervical cancer, we found that HPV genotypes distribution differs slightly from published data. HPV16 and HPV18 represented respectively 32 and 5% of all cases without any difference by grade in our population sample. Although HPV16 remained the most frequent genotype in invasive cancers, HPV31, HPV33 and HPV52 had the highest prevalence in our population.

We also showed the feasibility of HPV genotyping from paraffin embedded blocks in a tropical region. All previous work on HPV epidemiology in Guadeloupe were performed on fresh samples [14, 19, 20]. Archiving paraffin embedded blocks in tropical regions with humid weather, is known to hamper DNA extraction and amplification during HPV genotyping [21]. In our study, the samples were kept in designated areas before de-archiving. DNA was available for all the samples, but the lack of sufficient tumor material or the cutting of the tumor area remained uncontrolled factors. Our results nevertheless corroborates existing data; thus showing that this technique is robust with a good processing-performance (94%) [16].

In terms of HR-HPV genotype distribution, we found a higher prevalence of HR-HPV genotypes excluding HPV16 and HPV18: 60.6% (95% CI =53–68%), confirming the importance of non-HPV16/18 types on our territory. The most frequent HR-HPV types involved in our samples excluding HPV16, were HPV31, HPV33 and HPV52. Our results coincide with a previous study conducted on healthy women attending cervical cancer screening in Guadeloupe. A lower frequency of HPV16 or HPV18 (7.3%) was reported compared to other HR-HPV (28.8%) considered as a pooled category [14]. In our study on precancerous lesions and invasive cervical cancers, the overall prevalence of HPV16 was 32%, lower than the worldwide estimate of 54.4%. Non HPV16 and 18 accounted for 63% of all cases in our population. We were also able to identify the most frequent combinations of HR-HPV involved, and notably highlight the numerous occurrences of the two pairs: HPV31–33 and HPV31–52.

These results show similarities with other Caribbean and Central American countries. In Mexico, the study by Gallegos-Bolaños et al. reported that, with respect to the HPV16/18 combination, HPV16 was present at the third position after HPV51 and 52 but before HPV33 while HPV 18 was far behind [22]. In Curacao, Hooi et al. showed that despite being the first HPV genotypes in cervical cancer in respectively 38.5 and 13.5% of cases, HPV16 and HPV18 prevalence were lower compared with the world prevalence [23].

A meta-analysis and a French study conducted on women with invasive cervical cancer and high-grade cervical lesions showed that HPV16 and HPV18 were the most common in all continents [10, 15]. In the meta-analysis by Smith et al., few data on HPV and cervical lesions were available for the Caribbean region besides Jamaica, and thus may not reflect the HPV distribution in the region.

The role of multi-infection in aggravation of cervical lesions has already been highlighted in several studies. The sequence of primary infection/clearance/re-infection or over-infection seems to be decisive in the evolution of intra-epithelial lesions towards cancer. In a Brazilian cohort study, Trottier et al. suggested that the cumulative number of HPV genotypes were relevant in the evolution of cervical lesions [24]. Likewise, Hajia et al. demonstrated a linear relationship with histological grade [25]. On the contrary, in several studies, the severity of the lesion at diagnosis was not associated with multiple infection [26–29].

We found a higher frequency of 2–3 viruses in patients with invasive cancer cases compared to CIN 2/3 cases. We also documented the distribution of multiple infections with combined high-risk genotypes (i.e. 2 or more combined HR-HPV genotypes) and found that HPV31 and HPV52 were associated with CIN 2/3, whereas HPV35 and HPV33 did not show differences between CIN 2/3 and cancer. Our data on multiple infections were consistent with the results from Soto-De Léon et al., in Colombian women [30] but in opposition with data from China [26], India [31] Jamaica, Tobago and the US [32] where single infections prevailed.

Although our study was not designed to investigate the natural history of HPV infections on cervical cancer, our results provide valuable insight on the frequency of certain HR-HPV genotypes specific to the Afro-Caribbean population. In our sample, HPV31–33 seems to have similar frequency than HPV16. HPV16, HPV31, HPV33 and HPV52 are all in a same group of alpha papilloma virus (A9).

The high prevalence of the non-HPV16 types could be explained by a synergy between them as suggested by other studies [24]. Thus, Soto-De Léon et al. showed that the HPV genotype, its viral load and the kind of tissue impacted, have a role in the clearance stage [30]. Likewise, Trottier et al. found that HR-HPV types other than HPV16 could also interact and increase first risks observed with single-type infections.

Our findings will also provide insights for the ongoing implementation of the national cervical screening program in Guadeloupe. Early detection of high-grade lesions is expected with the program, resulting in a reduction of age at diagnosis. The identification of the involved HPV genotypes, and the delivery of comprehensive information could reduce the mistrust toward HPV vaccine and improve coverage. HPV vaccines are produced to prevent cervical cancer and the latest nonavalent vaccine targeting HPV16–18–06-11-31-33-45-52-58, is available since 2016. Although the potential immunization coverage of this vaccine could be considered appropriate for 75% of  CIN 2/3 patients and 69% of cancer patients in our population, it remains slightly lower compared to data from mainland France (81 and 90% respectively).

The limitations of our study are firstly, our small sample size. Nevertheless, the cases included were representative of all the cancer cases diagnosed in Guadeloupe for the study period and recorded through the population-based cancer registry. The volume of biological specimens was sometimes insufficient to perform adequately HPV genotyping, resulting in the exclusion of subjects for certain analyses. The removal of these subjects however is thought to be at random thus the possibility of attrition bias is kept to a minimum. Nevertheless, the method was carried out in good conditions and the results obtained for a large proportion of our blocks prove the feasibility of using FFPE block for the detection of HPV despite the humid tropical climate.

## Conclusion

This study describes for the first time the distribution of HPV genotypes in women from Guadeloupe with cervical cancer and CIN2/3 lesions. The most frequent genotypes were HPV31, HPV33, HPV16 and HPV52. Therefore, the coverage of the nine-valent HPV vaccine is adequate despite being lower than in European populations. Further studies are needed to understand the role of the multiple concurrent infections observed in our Afro-Caribbean population.

## Data Availability

The dataset generated during the current study are not publicly available but are available from the corresponding author on reasonable request.
